# Receipt of pharmacotherapy for alcohol use disorder by justice-involved women in the Veterans Health Administration

**DOI:** 10.1186/s13722-018-0129-x

**Published:** 2019-01-03

**Authors:** Emmeline Taylor, Christine Timko, Alex H. S. Harris, Mengfei Yu, Andrea K. Finlay

**Affiliations:** 1 Department of Veterans Affairs Health Care System, Center for Innovation to Implementation, Palo Alto, CA 94304 USA; 2 Department of Psychiatry and Behavioral Sciences, Stanford University School of Medicine, Stanford, CA 94305 USA; 3 Department of Surgery, Stanford University School of Medicine, Stanford, CA 94305 USA; 4 Department of Veterans Affairs, National Center on Homelessness Among Veterans, Menlo Park, CA 94025 USA; 5 795 Willow Road (MPD-152), Menlo Park, CA 94025 USA

**Keywords:** Alcohol use disorder, Criminal justice, Women, Veterans, Pharmacotherapy, United States Department of Veterans Affairs

## Abstract

**Background:**

Alcohol use disorder (AUD) and unhealthy drinking are prevalent among women involved in the criminal justice system and women military veterans. Pharmacotherapy—including naltrexone, topiramate, acamprosate, and disulfiram—for AUD is one form of effective treatment that is associated with better health and criminal justice outcomes. The current study examined the association of justice involvement with receipt of pharmacotherapy for AUD, as well as other patient factors that may explain variation in receipt of pharmacotherapy for AUD among women veterans who receive care at Veterans Health Administration (VHA) facilities.

**Methods:**

Using national VHA clinical records, we examined all women VHA patients who received an AUD diagnosis during an outpatient or inpatient visit in fiscal years (FY) 2014–2017. We compared patient characteristics by justice status, defined as contact with one of the VHA’s justice outreach programs, and used a mixed-effects logistic regression model to test whether justice involvement was independently associated with odds of receiving pharmacotherapy for AUD.

**Results:**

Of 10,511 women veterans diagnosed with AUD in FY2017, 852 (8%) met our definition of justice-involved. Since FY2014, the percentage of women veterans who received pharmacotherapy for AUD increased (14–21%). Women justice-involved veterans and those who were homeless had higher odds of receiving pharmacotherapy for AUD (OR 1.29, CI 1.15–1.45; OR 1.35, CI 1.25–1.47). Women veterans age 55 or older or who were African-American had lower odds of receiving pharmacotherapy (OR 0.74, CI 0.67–0.82; OR 0.73, CI 0.68–0.79).

**Conclusions:**

While women involved in the criminal justice system face many barriers to accessing health and social services, women justice-involved veterans had higher odds of receiving pharmacotherapy for AUD at VHA facilities compared to women veterans with no justice involvement. Legal mandates and supportive programming directed towards veterans in the criminal justice system may explain the higher rate of receipt of pharmacotherapy observed among justice-involved women veterans. Women veterans who are homeless may also have more opportunities to access and use pharmacotherapy for AUD compared to their housed counterparts.

## Background

Alcohol use can contribute to women becoming involved in the criminal justice system. Women under the influence of alcohol are at greater risk for committing a violent crime [1], and about a quarter of women on probation, in jail, or in state prisons, and 15% of women in federal prisons, were using alcohol when they committed their crimes [2]. Additionally, alcohol use disorder (AUD) is more prevalent among women involved in the criminal justice system compared to women in the general population. In a nationally representative sample of jail inmates in the U.S., 37% of women self-reported alcohol abuse or dependence in the year prior to their jail admission, whereas among women in the general population, approximately 4% self-reported past year alcohol abuse or dependence [3, 4].

Unhealthy alcohol use is also prevalent among women veterans, putting them at risk of criminal justice involvement. Past research found the prevalence for alcohol misuse among women Department of Veterans Affairs (VA) patients to be 27%, and 32% among women veteran non-VA users [5]. Additionally, women who have experienced trauma or who have posttraumatic stress disorder (PTSD) are at an elevated risk for developing AUD [6]. When asked about factors relating to their substance use, women veterans frequently cited childhood trauma, as well as military sexual assault and harassment [7]. Similar to the general population of incarcerated women, 36% of incarcerated women veterans self-reported having an AUD during an in-person visit with outreach staff who work in prisons [8], and 41% of women veterans who received outreach while in jail or court were diagnosed with AUD within a year of that outreach [9].

Pharmacotherapy for AUD (i.e. medications) is one form of effective treatment that has been associated with a decrease in health care costs, alcohol use, and recidivism [10–16]. The American Psychiatric Association and Department of Defense (DoD)/Veterans Health Administration (VHA) practice guidelines for the use of pharmacotherapy for patients with AUD list naltrexone, acamprosate, disulfiram, and topiramate as pharmacological treatment options [17, 18]. Naltrexone, acamprosate, and topiramate have been shown to be effective at increasing the number of days of alcohol abstinence and reducing heavy drinking days [12–16], and disulfiram, in open-label trials, has been found effective in supporting abstinence [19]. For veterans who are eligible for treatment at VHA facilities, pharmacotherapy for AUD is mandated to be available and considered when indicated [20].

While access to pharmacotherapy for AUD is a consensus standard of care, women with criminal justice contact face many barriers to accessing this substance use disorder (SUD) treatment modality. Past research found that women in jail who had a SUD were more likely to report a need for housing, mental health counseling, job training, medical care, and parenting assistance which could limit their ability to access and attend SUD treatment [21]. Additionally, women in the correctional system often present with other complex mental health problems, and treatment for SUDs can be easily overlooked [22]. Stigma based on substance use and incarceration is a further challenge for women with criminal justice contact, and often restricts their access to many health and social services [23]. Many of these challenges faced by justice-involved women veterans are challenges that can be addressed through integrated care provided by the VHA health care system. However, unknown is whether justice contact is a factor that limits women veterans’ access to pharmacotherapy for AUD. The primary aim of the current study was to examine the association of justice involvement with receipt of pharmacotherapy for AUD, adjusting for other patient and facility factors. The secondary aim was to examine other patient factors that may explain variation in receipt of pharmacotherapy for AUD among women veterans who receive care at VHA facilities.

## Methods

Using national VHA clinical records and administrative data, we conducted an observational study of all women VHA patients who received an AUD diagnosis (excluding in remission) during an outpatient or inpatient visit in fiscal years (FY) 2014–2017. AUD diagnosis was determined using International Classifications of Diseases (ICD)-9th and 10th Edition-CM codes, based on the American Society of Addiction Medicine specifications [24]. This study was approved by the VA Palo Alto Health Care System and the Institutional Review Board at Stanford University.

### Measures

#### Patient characteristics

Demographic variables included *gender*, *age*, *ethnicity/race* (non-Hispanic African-American, non-Hispanic White, and Other, for which all other races and ethnicities were collapsed, based on the Bureau of Census categories), *marital status* (single, married, separated/divorced, widowed), urban or rural *residence* (living in an urban city or rural area), and *homeless* status (drawn from a homeless indicator variable, housing clinic stops and residential codes, and ICD-9th and 10th Edition codes for housing and homelessness). Military characteristics included *service*-*connected disability rating,* which reflects a VHA-determined percent coverage for disabilities caused by illness or injury occurring during or aggravated by military service. The higher the service-connected disability rating percentage, the more compensation the veteran receives. Demographic and military characteristics were coded from records collected the same day the veteran received her AUD diagnosis during the FYs of interest, or from the next available record. Patient health characteristics included *co*-*occurring psychiatric disorder* (depressive disorders, post-traumatic stress disorder, anxiety disorders, bipolar disorder, schizophrenia, other psychosis, or personality disorders), *co*-*occurring substance use disorder* (SUD; opioid use disorder, cocaine use disorder, amphetamine use disorder, cannabis use disorder, sedative use disorder, or other drug use disorders) and the *Charlson*-*Deyo Comorbidity Index,* a sum of up to 17 comorbid medical diagnoses, such as HIV, liver disease, diabetes, and congestive heart failure [25]. These health characteristics were coded from records from each FY in which the veteran received an AUD diagnosis.

#### Justice involvement

A veteran was considered justice-involved if she had a clinic code indicating contact with the Health Care for Reentry Veterans (HCRV) program (591 code), which provides outreach to veterans exiting prison, or the Veterans Justice Outreach (VJO) program (592 code), which provides outreach in other justice system settings, primarily jails and courts, in the year prior to or during any of the FYs between 2014 and 2017 [26]. Women who did not have contact with either VHA justice outreach program were coded as not justice-involved.

#### Outcome

*Receipt* of pharmacotherapy for AUD was defined as filling at least one prescription for naltrexone, acamprosate, topiramate, or disulfiram as indicated in VHA pharmacy records during the FY period they received an AUD diagnosis. Receipt of pharmacotherapy for AUD is a performance measure endorsed by the American Society of Addiction Medicine [27].

### Analyses

For FY 2017, we examined descriptive statistics of women veterans’ characteristics, stratified by justice-involved status, and tested for group differences using Chi square analyses. Cases with missing data (n = 236, 2%) were excluded from the Chi square analyses. The rate of receipt of pharmacotherapy for AUD was calculated separately for each FY as the number of women veterans who received pharmacotherapy for AUD within 1-year of diagnosis divided by number of women veterans diagnosed with AUD overall and by medication type. A mixed-effects logistic regression model using SAS PROC GLIMMIX was used to test the association between justice-involved status and receipt of pharmacotherapy for AUD across all FYs examined. All women veterans’ characteristics and FY indicators were simultaneously entered in the model as main effects to account for the demographic and time trend adjustments. Nested random effects were used to model a three-level hierarchical data structure: patients within years within facilities. Cases with missing data (n = 619, 1%) were excluded from the mixed-effects model.

## Results

### Characteristics of women veterans diagnosed with AUD by justice-involved status

Of 10,511 VHA women patients diagnosed with AUD in FY2017, 852 (8%) had contact with one of the VHA’s justice outreach programs for individuals in the criminal justice system. Characteristics of women veterans with AUD in FY2017 are displayed by justice-involved status in Table 1. Due to similarities across FYs examined, we only report demographics for FY2017. Justice-involved veterans with AUD were younger (p < .01), and more likely to be White (p < .01) compared to their not justice-involved counterparts. Fewer justice-involved veterans with AUD were married compared to those not justice-involved (p < .01). Among veterans with AUD who were justice-involved, 42% were homeless compared to 17% of those not justice-involved (p < .01). Fully, 95% of veterans with justice involvement had a co-occurring mental health disorder diagnosis compared to 89% of those not justice-involved (p < .01), and 60% of justice-involved veterans were diagnosed with a co-occurring SUD compared to 31% of veterans with no known justice involvement (p < .01).

**Table 1:** Characteristics of women veterans diagnosed with alcohol use disorder in fiscal year 2017 by justice-involved status

Characteristic	Justice-involved N (%)	Not justice-involved N (%)
Age^a^		
35 and under	218 (27)	1554 (16)
35–44	191 (23)	1863 (20)
45–54	225 (28)	2652 (28)
55+	184 (22)	3388 (36)
Race/ethnicity^a^		
White	497 (61)	5124 (54)
African-American	223 (27)	3121 (33)
Other	98 (12)	1212 (13)
Marital status^a^		
Married	137 (17)	2194 (23)
Single	330 (40)	3287 (35)
Separated/divorced	333 (41)	3654 (39)
Widowed	18 (2)	322 (3)
Urban (versus rural)	640 (78)	7275 (77)
Homeless^a^	344 (42)	1598 (17)
Service connection		
0–49%	175 (21)	2032 (21)
50–100%	346 (42)	4066 (43)
Co-occurring SUD^a^	493 (60)	2887 (31)
Mental health diagnosis^a^	777 (95)	8431 (89)
Deyo Comorbidity Index^a^		
0	692 (85)	8273 (87)
1	98 (12)	787 (8)
2	17 (2)	195 (2)
3+	11 (1)	202 (2)

N = 10,275. Cases with missing data (n = 236, 2%) were excluded from the Chi square test

*SUD* substance use disorder

^a^Chi square test significant at p < .01

### Receipt of pharmacotherapy for AUD

The percentage of women veterans, both justice- and non-justice-involved, who received pharmacotherapy for AUD increased from 14% in FY2014 to 21% in FY2017 (p < .0001). Additionally, of women veterans who had contact with a VHA justice outreach program, 20% received pharmacotherapy for AUD in FY2014 compared to 31% in FY2017 (p < .0001). The mixed-effects logistic regression model indicated that women veterans with justice involvement had higher odds of receiving pharmacotherapy for AUD compared to women veterans not justice-involved (p < .0001, Table 2). Additionally, compared to women veterans less than 35 years old, those age 35–54 had higher odds of receiving pharmacotherapy for AUD whereas those age 55 or older had lower odds (p < .0001, Table 2). African-American women veterans had lower odds than White women veterans of receiving pharmacotherapy (p < .0001, Table 2). Women veterans who were homeless, had a co-occurring SUD, or another mental health diagnosis also had higher odds of receiving pharmacotherapy for AUD (p < .0001, Table 2).

**Table 2:** Characteristics associated with receipt of pharmacotherapy for alcohol use disorder among women veterans in fiscal years 2014–2017

Characteristic	OR	95% CI
Justice-involved	1.29***	1.15–1.45
Age (ref: < 35)		
Ages 35–44	1.31***	1.19–1.45
Ages 45–54	1.16***	1.06–1.27
Ages 55+	0.74***	0.67–0.82
Race/Ethnicity (ref: White)		
African-American	0.73***	0.68–0.79
Other	0.98	0.85–1.12
Single (ref: married)	0.90*	0.83–0.98
Rural residence (ref: urban)	0.94	0.86–1.02
Homeless (ref: housed)	1.35***	1.25–1.47
Service-connected disability rating (ref: none)		
< 50%	1.01	0.92–1.11
≥ 50%	1.32***	1.22–1.42
Mental health diagnosis	4.06***	3.45–4.78
Co-occurring SUD diagnosis	1.50***	1.41–1.61
Deyo Comorbidity Index	1.08*	1.00–1.17

*N* 44,090. Cases with missing data (n = 619; 1%) were excluded from the mixed-effects logistic regression model

*OR* odds ratio; *CI* confidence interval; *SUD* substance use disorder

*p < .05; ***p < .0001

## Discussion

The primary aims of this study were to examine the association of justice involvement with receipt of pharmacotherapy for AUD and examine other patient factors that may explain variation in receipt of pharmacotherapy for AUD among women veterans who receive care at VHA facilities. We found that women veterans who had contact with a VHA justice outreach program had higher odds of receiving pharmacotherapy for AUD compared to women veterans with no known justice involvement, even after adjusting for other patient characteristics. Programming and legal mandates for veterans with criminal justice system contact may partially explain the higher rate of pharmacotherapy receipt. Some veterans with criminal justice involvement participate in treatment courts and others are on probation or parole. Through those systems, they may have a requirement to seek AUD treatment which could include pharmacotherapy. Although we could not identify which veterans in our sample had a legal mandate for treatment, prior work with the general population exiting prison found that legal supervision and court mandates were associated with participation and adherence in SUD treatment, including pharmacotherapy [28, 29].

The VHA’s justice outreach programs, which are designed to assess the treatment needs of veterans with criminal justice involvement and connect them with appropriate services, may also explain the higher rate of receipt of pharmacotherapy for AUD. Outreach workers from these programs support veterans with tasks as diverse as helping them complete benefits paperwork, find housing, and schedule health care appointments. Connecting justice-involved veterans with SUD treatment, which may include pharmacotherapy, is one of the priorities of the program, and most participants enter and engage in SUD treatment [9]. Justice-involved women veterans with SUD have an average of 31 SUD-specific outpatient visits a year compared to an average of 15.2 visits among women veterans with SUD in general, with more visits potentially increasing their likelihood of considering or receiving one of these AUD medications [9, 30]. Given the substantial challenges justice-involved populations face, such as stigma and difficulty finding employment and housing [31, 32], outreach efforts that smooth these barriers can support veterans in their ability to then focus on their health and treatment for AUD. Although women veterans who had contact with a VHA justice outreach program were more likely to receive pharmacotherapy for AUD than women with no known justice involvement, the overall rate of receipt for justice-involved and non-justice-involved women veterans in this study was low compared to rate of receipt for pharmacotherapies for other mental health disorders and non-pharmacological treatment for AUD among women veterans [30, 33]. Additionally, prior research indicates SUD is a risk factor for criminal justice system contact among women, suggesting that women veterans who don’t receive pharmacotherapy for AUD, or an equally as effective treatment, may be increasing their risk for contact with the criminal justice system, whether or not they had previous involvement [34].

Other characteristics of women veterans were also associated with receipt of pharmacotherapy for AUD. Women veterans who were homeless had increased odds of receiving pharmacotherapy for AUD. One possible explanation for the higher rate of receipt may be VHA’s homeless veterans’ initiatives. Similar to the VHA’s justice outreach programs, VHA’s homeless initiatives and supported housing programs help connect homeless veterans to housing solutions and medical, mental health, and SUD treatment which can include pharmacotherapy for AUD. Prior research showed homeless women veterans were more likely than men to enter a VHA supported housing program, and reported less substance use over the subsequent year [35], suggesting that they may be engaging in some form of SUD treatment. Additionally, homeless women veterans reported significantly more mental health outpatient visits in the 3 months following their contact with the supported housing program compared to the 3 months prior [36]. These visits may provide more opportunity for homeless women with AUD to engage in pharmacological treatment. Among homeless individuals who are currently justice-involved, they are more likely to have past criminal justice system involvement and substance use problems when compared to their non-homeless counterparts [37]. This suggests that justice-involved women veterans with AUD who are homeless may be at an increased risk for recidivism, thus providing pharmacotherapy for AUD may be especially beneficial to this subpopulation of women veterans.

African-American women veterans were less likely to receive pharmacotherapy for AUD, consistent with prior research indicating that African-American VHA patients overall are less likely to receive pharmacotherapy for AUD compared to White patients [38]. Additionally, previous studies found lower utilization and completion rates of AUD treatment by African-American adults, which were linked to higher perceived mental health stigma in the community and other factors related to treatment experience [39, 40]. African-American women veterans may be less likely to engage in AUD treatment more broadly, not just pharmacotherapy. Additionally, when examining VHA mental health care utilization, African-American women veterans were more likely to use women-only mental health treatment settings and expressed lower gender-related comfort with VHA mental health treatment, indicating that gender-specialized treatment may be an important care facilitator when trying to connect African-American women veterans with AUD care [41]. In the criminal justice setting, African-American women are more than 7 times as likely to be incarcerated compared to White women [42]. This evidence suggests that it is important that the VHA’s justice outreach programs connect African-American women veterans with AUD who have criminal justice system contact to pharmacotherapy. This will help lessen any AUD treatment disparities that may exist for justice-involved women veterans of color, and may help reduce recidivism among this group of women veterans.

Women veterans age 55 and older were less likely to receive pharmacotherapy for AUD than younger women. In contrast, past research has found that older adult women veterans use VHA mental health and SUD outpatient care more frequently than their younger counterparts [30]. One possible explanation for the lower rate of receipt of medication for AUD could be that older women or their prescribers are less likely to consider pharmacotherapy specifically [43]. Reasons for this may be that older women likely take other prescriptions causing concern about medication interactions, and compared to younger adults, older adults appear to have less severe AUD [44–46].

### Limitations

There are several limitations to our study, particularly because we examined secondary data. First, as our sample was limited to women veterans using VHA-services, women veterans receiving pharmacotherapy for AUD outside of VHA were not covered by the dataset. Second, due to our definition of justice involvement, there may have been veterans with criminal justice contact that were misclassified as non-justice-involved in this study. However, across the general population of veterans, less than 3% are justice involved [26], and it is likely that only a small number of veterans were misclassified in this study. Third, this study examined receipt of pharmacotherapy, and therefore little can be said regarding medication adherence or use of those who were prescribed pharmacotherapy for AUD. Lastly, we were unable to examine other treatments for SUD or mental health that veterans in our sample may have been receiving. As discussed, it is possible that other SUD or mental health treatment could increase the likelihood that a veteran would receive pharmacotherapy for AUD.

## Conclusions

This study found that receipt of pharmacotherapy for AUD is increasing among women veterans generally, and among women veterans who have had contact with the VHA’s justice outreach programs specifically. Many barriers to AUD care experienced by justice-involved women are being addressed by the VHA’s integrated health care system, which helps to explain the higher odds of receiving pharmacotherapy for AUD among the women justice-involved veterans compared to their non-justice-involved counterparts. Women veterans experiencing homelessness were also more likely to receive pharmacotherapy for AUD; this finding may also be partially explained by the VHA’s integrated care model and support provided to homeless veterans. Generally, African-American and older women veterans had lower odds of receiving pharmacotherapy for AUD, suggesting that these women may have unique barriers to accessing or utilizing this effective treatment.

Legal mandates and supportive programming targeting veterans with criminal justice system contact may explain the higher rate of receipt of pharmacotherapy for AUD observed among justice-involved women veterans in our study. To better improve receipt and use of pharmacotherapy for AUD among women veterans with criminal justice involvement, we need to better understand their general treatment and service access and use. In the criminal justice and health field, future research, such as qualitative studies examining treatment and service access, preference, and entry among women veterans with criminal justice involvement may help identify barriers to and facilitators of accessing pharmacotherapy for AUD. Additionally, research with health care providers and facilities offering pharmacotherapy for AUD may help shed light on implementation strategies and facilitators that increase use of these medications among women veterans with criminal justice contact, and women veterans generally, who are interested in this treatment option.

## Abbreviations

AUD: alcohol use disorder
CI: confidence interval
DoD: Department of Defense
FY: fiscal year
HCRV: Health Care for Reentry Veterans
ICD: International Classifications of Diseases
PTSD: posttraumatic stress disorder
SUD: substance use disorder
VA: Veterans Affairs
VHA: Veterans Health Administration
VJO: Veterans Justice Outreach
